# Exploration on the Improvement of Cognitive Function and Inflammatory Response in Perimenopausal Patients with Mild Cognitive Impairment by Self-Prepared Ningshen Prescription

**DOI:** 10.1155/2022/4311031

**Published:** 2022-06-02

**Authors:** Wei Yang, Yumei Ye, Yan Cai, Guiyan Wang, Menghao Wang, Xiaodan Zhang

**Affiliations:** Department of TCM, Seventh People's of Shanghai University of TCM, Shanghai 200137, China

## Abstract

**Objective:**

To investigate the improvement of cognitive function and inflammatory response in perimenopausal patients with MCI by kidney-tonifying, blood-activating, and mind-nourishing.

**Methods:**

80 perimenopausal patients with MCI who met the diagnostic criteria were divided into a therapy group (*n* = 40) and a control group (*n* = 40) according to the treatment method. The control group was given nimodipine (Bayer Pharmaceuticals) 30 mg, 3 times/day orally, while the therapy group was given a decoction of self-prepared Ningshen prescription on the top of the control group (glossy privet fruit, mulberry, aizoon stonecrop, dan-shen root, tuber fleeceflower stem, cyperus rotundus, citron). Patients in the 2 groups were assessed on the MocA scale, ADL scale, and TCM symptom score before and after 2 months of treatment, respectively, to observe whether there was any change in the scale scores and in the levels of inflammatory factors (hs-CRP, Hcy, and IL-1*β*) Pre- and posttherapy in the 2 groups. Observe the improvement of clinical symptoms and their safety in both groups (liver and kidney function indicators such as ALT, AST and Cr, dizziness, headache, decrease in blood pressure, flushing, and gastrointestinal reactions).

**Results:**

The efficacy of the therapy group was better than that of the control group; the MocA scale and ADL scale scores improved and the TCM symptom score decreased in both groups posttherapy, with the MocA scale and ADL scale scores improving more and the TCM symptom score decreasing more in the therapy group compared with the control group during the same period (*p* < 0.05). The serum levels of hs-CRP, Hcy, and IL-1*β* decreased in both groups posttherapy, with the serum levels of hs-CRP, Hcy, and IL-1*β* decreasing more in the therapy group compared to the control group during the same period (*p* < 0.05). The difference in adverse events between the two groups was not statistically significant when compared by a chi-square test (*p* > 0.05). The differences in ALT, AST, and Cr levels between the control group and the treatment group before and after treatment were not significant (*p* > 0.05).

**Conclusion:**

Ning Shen prescription can effectively prevent the continued development of cognitive dysfunction in perimenopausal patients with MCI, delay its natural course, and can improve the patients' ability to perform daily activities and improve their TCM symptoms.

## 1. Foreword

Perimenopause is a period of physiological changes that every woman goes through around the time of menopause. During this period, some women experience menstrual disorders, sweating, irritability, and mild cognitive impairment such as difficulty concentrating and reduced learning and memory skills due to changes in ovarian function and reduced production of sex hormones [1, 2]. Patients with mild cognitive impairment (MCI) do not meet the diagnostic criteria for dementia in terms of severity of cognitive function but fall between normal aging of memory and dementia, which may eventually progress to Alzheimer's disease or other types of dementia, and are generally prevented and treated by medical researchers as a predementia stage [3]. In recent years, the incidence of various geriatric diseases, such as dementia, has been increasing due to the ageing of the population. Dementia and MCI, which are characterised by impairment of intelligence, pose a number of medical and social problems, particularly for family members, which are particularly acute in modern, fast-paced society [4, 5].

There is still a lack of authoritative statements on the pathogenesis of MCI, and the drugs that are more widely used clinically for treatment are those that mainly improve symptoms, such as cholinesterase inhibitor (acetylcholinesterase inhibitor, AchEI) and ionotropic glutamate receptor antagonist (N-methyl-D-aspartate receptor), but according to the available findings [6, 7], there is still no significant evidence on the efficacy of these drugs in intervening the conversion rate of MCI to dementia, and further studies are needed to confirm this. In recent years, with in-depth research on MCI, Chinese medicine has achieved good results in improving symptoms, delaying the disease, and improving patients' quality of life, and the investigation of its intrinsic mechanism has received a lot of attention from clinical workers [8, 9]. Cognitive disorder is the name of a modern medical disease, but Chinese medicine does not have an exact name for it, rather it is classified as a disease related to “dementia,” “forgetful” and other mental illnesses based on its clinical manifestations in memory, thinking and language [10]. Chinese medicine believes that the disease of good forgetfulness is located in the brain and its pathological changes are closely related to the heart, spleen, and kidney. In recent years, several studies [11, 12] have classified statistics on the TCM typology of MCI, and the typology of MCI is based on the common features of MCI such as kidney deficiency, liver depression, and phlegm and blood stasis, which brings new ideas to the treatment of MCI.

In this study, after collecting clinical data and collating the literature, we selected MCI patients with kidney deficiency and blood stasis as the study subjects to observe the clinical effectiveness and drug safety of treatment with Ning Shen prescription in MCI patients with kidney deficiency and blood stasis.

## 2. Information

### 2.1. General Data

80 cases of perimenopausal female MCI patients in our hospital from October 2020 to October 2021 were selected and divided into 40 cases each in the therapy group (Ning Shen prescription) and the control group (nimodipine treatment), and the general information of the 2 groups is compared in Table 1.

### 2.2. Diagnostic and Identification Criteria

#### 2.2.1. Western Medical Diagnosis

Diagnostic criteria for MCI in perimenopause were developed based on previously published literature related to diagnostic criteria for MCI [13], and perimenopausal characteristics [14]. (i) Women aged 40–60 years; (ii) self-reported memory loss or informed reports of memory loss; (iii) MOCA scale score <26, plus 1 point if the patient has less than or equal to 12 years of education; General Decline Scale (GDS) = 2-3 or Clinical Dementia Rating Scale (CDR) = 0.5; (iv) Normal daily living: score of <26 on the Ability to Perform Daily Living Scale (ADL); (v) whose cognitive decline has not yet met the diagnostic criteria for dementia.

#### 2.2.2. Diagnosis in Chinese Medicine

By reviewing the literature [15, 16], the diagnostic criteria for perimenopausal MCI of the kidney deficiency and blood stasis type were developed: (i) women aged 40–60 years; (ii) main symptoms: forgetfulness and memory loss; (iii) secondary symptoms: soreness and weakness of the waist and knees, tiredness and sleepiness, dizziness and tinnitus, heavy limbs, headache like a thorn or a pain that does not move, dark purple in the mouth and claws, and dry skin; and (iv) tongue and pulse: purple tongue or petechiae, dark veins under the tongue, fat tongue, greasy coating; sluggish or sunken pulse. The diagnosis can be made by having one of the main symptoms plus two of the secondary symptoms combined with the tongue and pulse.

### 2.3. Inclusion Criteria

Incluion criteria were as follows:

- Those who met the above diagnostic and identification criteria in Western medicine and Chinese medicine
- Those who were perimenopausal women aged 40∼60 years old and had no reproductive plans
- Those who met the Kupperman Index (KI) score ≥15 on the Modified KI Scale
- Those who did not receive other drugs or methods of treatment for cognitive impairment 4 weeks prior to this treatment

### 2.4. Exclusion Criteria

Exclusion criteria were as follows:

- Cognitive dysfunction caused by other diseases
- Those who were using drugs prohibited by the study and cannot stop
- Combination of serious primary diseases such as severe cardiovascular, hepatic, renal and hematopoietic system, psychiatric patients, or systemic diseases such as pain, fever, cough, surgery.
- Those with severe neurological deficits who could not cooperate with the physician to complete the relevant tests
- Alcohol and drug abusers; patients who had undergone surgery or medical conditions that affect pharmacokinetics such as gastrointestinal surgery or disease

### 2.5. Treatment

Those who met the inclusion criteria were randomly divided into the therapy group and the control group. Both groups were given conventional medication for the underlying disease, such as hypertension and diabetes mellitus patients with antihypertensive drugs and hypoglycemic drugs, respectively, and those without contraindications to aspirin were treated with aspirin 0.1 g, 1 time/day.

In the control group, nimodipine (Bayer Pharmaceuticals) 30 mg was given orally 3 times/day. In the therapy group, Ning Shen prescription was added to the control group with water decoction; 150 ml/time, 2 times/day, for 2 months. Ning Shen prescription consists of glossy privet fruit, mulberry, aizoon stonecrop, dan-shen root, tuber fleeceflower stem, cyperus rotundus, and citron.

### 2.6. Observation Indicators

- Patients were assessed on the Montreal Cognitive Assessment Scale (MoCA), ADL scale, and TCM symptom score pre- and posttherapy, respectively. The MocA scale was used to assess the cognitive function of patients pretherapy and after 12 weeks of therapy. The scale consists of 11 entries in 8 cognitive domains, specifically concentration, memory, language, computation and orientation, executive function, visual structure skills, and abstract thinking, with a total score of 30, and a score of 26 and above was considered normal. Patients' self-care ability was assessed by using the ADL scale, with a total score of 100, less than or equal to 19 being completely unable to take care of themselves, 20–39 being in need of greater assistance, 40–59 being partially in need of assistance, and 60 being basically able to take care of themselves. The higher the score, the better the patient's ability to take care of himself/herself.
- To observe whether there was any change in the scale scores pre- and posttherapy and whether there was any change in the levels of inflammatory factors (hs-CRP, IL-1*β*, and TNF-*α*) pre- and posttherapy in the 2 groups. Before and after therapy, 2 ml of elbow vein blood was drawn in the morning on an empty stomach, and the serum was centrifuged at 3000 r/min for 10 minutes to obtain the serum, which was labeled and stored at −80°C in the refrigerator for batch determination. The TNF-6 and IL-6 levels were measured by radioimmunoassay, and the kits were obtained from the Northern Immunological Reagent Institute of China Isotope Company. The level of hs-CRP in serum was measured by the immunoturbidimetric method by the professional laboratory staff of the Department of Laboratory Medicine of the hospital, using a Hitachi 7180 fully automatic biochemical analyzer and reagents from Shenzhen Jingmei Biotechnology Co.
- To observe the improvement of clinical symptoms and their safety in both groups (liver and kidney function indicators such as ALT, AST and Cr, dizziness, headache, decrease in blood pressure, flushing, and gastrointestinal reactions).
- Efficacy assessment criteria [17] were divided into effective, efficient, and ineffective; the MocA score was used as the main reference index and combined with the improvement of clinical symptoms to make a comprehensive evaluation, MOCA score = [(posttherapy score − pretherapy score)/pretherapy score] × 100%. ① Significant effect: MOCA score ≥40%, clinical symptoms and signs improved significantly. ② Effective: 20% ≤ MOCA ＜ 40%, clinical symptoms and signs have improved. ③ Invalid: MOCA score <20%, or even decreased, clinical symptoms and signs did not improve significantly, or even worsened.

### 2.7. Statistical Method

The data of this study were statistically analyzed using SPSS22.0 software. Count data were expressed as *n* (%) with *χ*^2^ test; measurement data were described as the mean ± standard deviation ($\bar{x}$ ± *s*) with *t*-test; differences were considered statistically significant at *p* < 0.05.

## 3. Results

### 3.1. Comparison of General Information of the Two Groups

According to Table 1, there were no significant differences in age distribution, age at menarche, proportion of menopause, education level, personal history, initial MocA, ADL, TCM symptom score and initial hs-CRP, Hcy and IL-1*β* levels in the 2 groups of MCI patients (*p* > 0.05).

### 3.2. Comparison of MocA Scale, ADL Scale Scores, and TCM Symptom Scores Pre- and Posttherapy between the Two Groups

**Figure 1:**
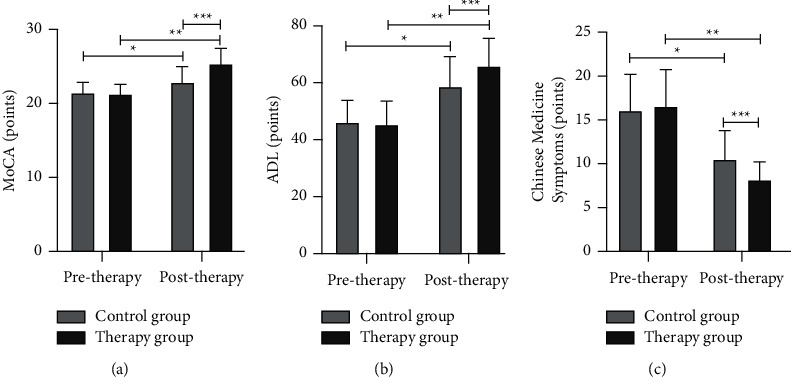
Comparison of MocA scale, ADL scale scores, and TCM symptom scores between the 2 groups ($\bar{x}$ ± *s*). (a) The MocA scale score, (b) the ADL scale score, and (c) the TCM symptom score. The special symbol ^*∗*^ is the difference between the control group pre- and posttherapy *p* < 0.05, ^*∗∗*^ is the difference between the therapy group pre- and posttherapy *p* < 0.05, and ^∗∗∗^ is the difference between the control group and the therapy group during the same period *p* < 0.05.

According to Figure 1, the differences in MocA scale, ADL scale scores, and TCM symptom scores between the 2 groups of MCI patients pretherapy were not significant (*p* > 0.05). Posttherapy, the MocA scale and ADL scale scores of the two groups improved, and the TCM syndrome points decreased (*p* < 0.05). Among them, the MocA scale and ADL scale scores improved more and the TCM syndrome points decreased more in the therapy group compared with the control group (*p* < 0.05).

### 3.3. Comparison of Inflammatory Factor Levels Pre- and Posttherapy between the Two Groups

**Figure 2:**
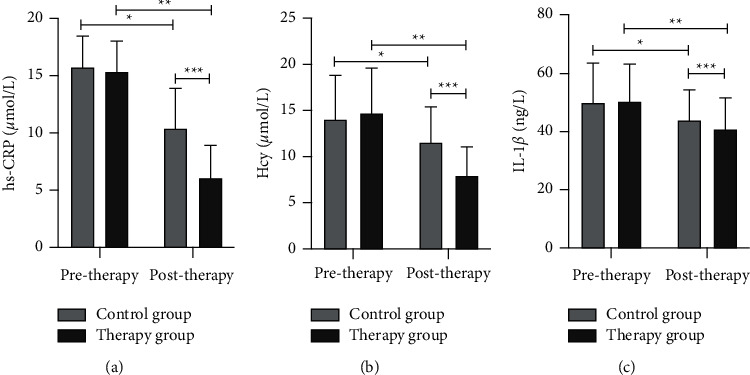
Comparison of serum hs-CRP, Hcy, and IL-1*β* levels in the 2 groups ($\bar{x}$ ± *s*). (a) The hs-CRP level, (b) the Hcy level, and (c) the IL-1*β* level. The special symbol ^*∗*^ is the difference between the control group pre- and posttherapy *p* < 0.05, ^*∗∗*^ is the difference between the therapy group pre- and posttherapy *p* < 0.05, and ^∗∗∗^ is the difference between the control group and the therapy group during the same period *p* < 0.05.

According to Figure 2, the differences in the serum levels of hs-CRP, Hcy, and IL-1*β* between the 2 groups of MCI patients pretherapy were not significant (*p* > 0.05). Posttherapy, the serum levels of hs-CRP, Hcy and IL-1*β* decreased in both groups (*p* < 0.05); the serum levels of hs-CRP, Hcy and IL-1*β* decreased more in the therapy group compared with the control group (*p* < 0.05).

### 3.4. Comparison of the Clinical Efficacy of the Two Groups

**Figure 3:**
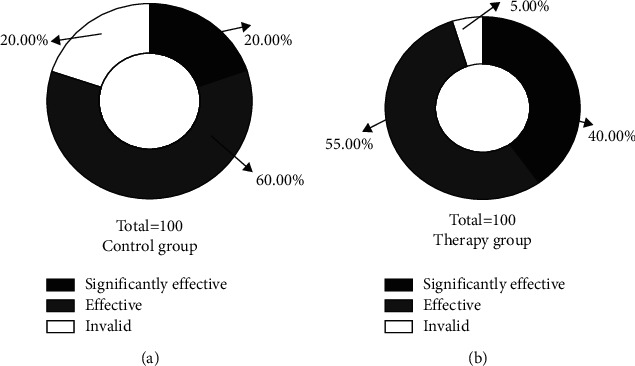
Comparison of clinical outcomes between the 2 groups (n, %). (a) The distribution of efficacy in the control group and (b) the distribution of efficacy in the therapy group.

According to Figure 3, the apparent, effective, and null rates of MCI patients in the control group were 20.00%, 60.00%, and 20.00%, respectively, while the apparent, effective, and null rates of MCI patients in the therapy group were 40.00%, 55.00%, and 5.00%, respectively. The overall effective rate of treatment was found to be better than that of the control group (*p* < 0.05).

### 3.5. Comparison of Adverse Events between the Two Groups

**Figure 4:**
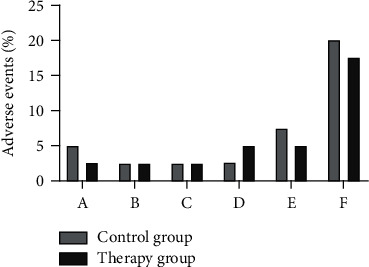
Comparison of adverse events in 2 groups (n, %). *Note.* In the graph, A indicates dizziness, B indicates headache, C indicates decreased blood pressure, D indicates flushed face, E indicates gastrointestinal reactions, and F indicates total incidence of adverse events.

The incidence of adverse events such as dizziness, headache, decreased blood pressure, flushed face, and gastrointestinal reactions during treatment in the control group of MCI patients was 5.00%, 2.50%, 2.50%, 2.50%, 2.50%, and 7.50%, respectively. The incidence of adverse events such as dizziness, headache, decreased blood pressure, flushed face, and gastrointestinal reactions during treatment in the MCI patients in the therapy group was 2.50%, 2.50%, 2.50%, 5.00%, and 5.00%, respectively. According to Figure 4, the overall incidence of adverse events such as dizziness, headache, decreased blood pressure, flushed face, and gastrointestinal reactions during treatment was not significantly different between the two groups of MCI patients (*p* > 0.05).

### 3.6. Comparison of Treatment Safety between the Two Groups

**Figure 5:**
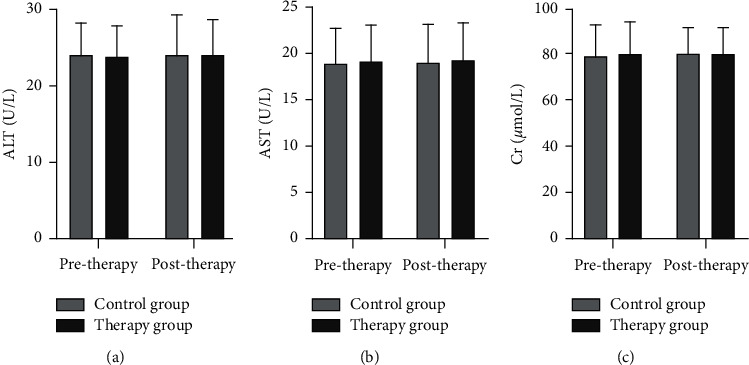
Comparison of treatment safety between the 2 groups ($\bar{x}$ ± *s*). (a) The serum ALT level, (b) the serum AST level, and (c) the serum Cr level.

According to Figure 5, the differences in ALT, AST, and Cr levels between the 2 groups of MCI patients before and after treatment were not significant (*p* > 0.05). The differences in ALT, AST, and Cr levels between the control group and the treatment group before and after treatment were not significant (*p* > 0.05).

## 4. Discussion

With the improvement of modern quality of life and medical care and the increasing ageing of society, the number of patients with Alzheimer's disease (AD) is increasing every year. AD is the most common cause of death in the elderly after tumors, cardiovascular diseases, stroke, and other diseases, which brings great burden and challenges to countless patients, families, and society [18]. Among the factors associated with the onset of AD, age, genetics, gender, and endocrine metabolism are the most predominant; the incidence of DA increases with age, and the clinical presentation differs between men and women, with a preference for women [19]. In addition, some studies [20, 21] have found that cognitive function is much more impaired in women than in men at the same stage of AD, which may be related to abnormal estrogen levels in peri- or postmenopausal women. MCI is often seen as a pre-AD state and a high risk factor for developing AD. Therefore, effective interventions for perimenopausal patients in the pre-AD state to improve symptoms and prevent or delay the onset of AD as much as possible is one of the current hot topics of research.

Western drugs such as brain cell activators and cerebral vasodilators are currently used in clinical work for the treatment of MCI, with average overall effects. In recent years, with the continuous development of Chinese medicine in China, herbal treatment has gained great achievements in many fields and has certain advantages in the clinical treatment of MCI [22]. According to the Chinese medical knowledge of the pathogenesis of MCI, the disease is located in the brain and is closely related to the kidney. The loss of kidney qi, insufficient qi and blood, and internal stagnation of stagnant blood cause the brain marrow and head orifices to lose moistening, and gradually the marrow sea is not filled, resulting in the loss of the use of the mental organ and causing the disease [23, 24]. In this study, treatment with the self-prepared Ning Shen prescription showed that the treatment group showed significantly higher improvements in cognitive ability, activities of daily living, inflammatory factor levels, and TCM symptom scores than the control group after treatment. The whole formula works together to nourish the liver and kidney, and to calm the mind. It also enriches the marrow to enable the brain to control the mental thinking and visceral functions of the body, which in turn helps to improve the symptoms of MCI. In addition, when the essence in the kidney is full, the marrow sea is nourished and the marrow is full, the brain spirit can effectively control the movement of the limbs, which helps to improve many symptoms of MCI. Modern pharmacological studies have confirmed that the liver and kidney tonics such as glossy privet fruit [25] and mulberry [26] can promote Bcl-2 gene expression in the body, reduce neuronal apoptosis, and improve blood viscosity and microcirculation to enhance blood flow to brain tissue and improve central nervous system function. Modern pharmacological research [27] found that Danshen contains tanshinone I and cryptotanshinone and other quinones, which can dilate coronary arteries and peripheral blood vessels and increase coronary blood flow, and its combined use with Panax ginseng has the functions of delaying brain aging and protecting nerve cells. In this study group, we combined Western medicine treatment with Chinese medicine diagnosis theory to treat perimenopausal women with MCI by Ning Shen prescription. The combination of Chinese and Western medicine worked in synergy, resulting in more significant improvements in cognitive function, quality of life, symptomatology, and inflammatory response in the therapy group. In addition, there were no patients with serious significant adverse reactions in either group throughout the clinical observation trial. The values of ALT, AST, and Cr in the therapy and control groups before and after the whole trial were not significantly different (*p* > 0.05). The statistical results basically indicated that the treatment and control groups had no serious effects on the liver and kidney functions of the patients in the short term and had a certain degree of safety.

In conclusion, the combination of Ning Shen prescription with western medicine can significantly improve the cognitive function, quality of life, and symptoms of perimenopausal female MCI patients, and the efficacy of Chinese medicine symptoms can be improved. There were no uncomfortable symptoms related to Chinese medicine during the study, no serious adverse events, and no significant effects on the liver and kidney functions of the patients in the short term, so the safety profiles were all excellent.

## Figures and Tables

**Table 1:** Comparison of general data between the two groups.

Data		Control group (*n* = 40)	Therapy group (*n* = 40)	*t* or *χ*^2^ value	*p* value
Age distribution (n, %)	40∼45 years	9 (22.50)	7 (17.50)	0.825	0.843
	46∼50 years	14 (35.00)	12 (30.00)		
	51∼55 years	13 (32.50)	16 (40.00)		
	56∼60 years	4 (10.00)	5 (12.50)		
Age at menarche (years, $\bar{x}$ ± s)		15.12 ± 1.37	14.89 ± 1.43	0.735	0.465
Duration of disease (months, $\bar{x}$ ± s)		4.03 ± 0.91	4.06 ± 0.93	0.146	0.884
Menopause (*n*, %)		9 (22.50)	7 (17.50)	0.313	0.576
Level of education (*n*, %)	Illiterate	4 (10.00)	6 (15.00)	1.376	0.711
	Primary school	13 (32.50)	12 (30.00)		
	Lower secondary	16 (40.00)	18 (45.00)		
	High school and above	7 (17.50)	4 (10.00)		
Personal history (*n*, %)	Smoking	9 (22.50)	11 (27.50)	0.267	0.606
	Alcohol consumption	7 (17.50)	10 (25.00)	0.672	0.412
MocA (points, $\bar{x}$ ± s)		21.32 ± 1.34	21.51 ± 1.43	0.613	0.542
ADL (points, $\bar{x}$ ± s)		45.51 ± 8.27	46.27 ± 7.87	0.421	0.675
TCM symptom score (points, $\bar{x}$ ± s)		16.23 ± 4.10	16.57 ± 4.23	0.365	0.716
hs-CRP (*μ*mol/L, $\bar{x}$ ± s)		15.87 ± 2.63	15.42 ± 2.66	0.761	0.449
Hcy (*μ*mol/L, $\bar{x}$ ± s)		14.21 ± 4.69	14.87 ± 4.76	0.625	0.534
IL-1*β* (ng/L, $\bar{x}$ ± s)		50.22 ± 13.18	50.46 ± 12.69	0.083	0.934
ALT (U/L, $\bar{x}$ ± s)		24.23 ± 4.07	23.95 ± 3.89	0.315	0.754
AST (U/L, $\bar{x}$ ± s)		19.08 ± 3.72	19.14 ± 4.03	0.069	0.945
Cr (*μ*mol/L, $\bar{x}$ ± s)		80.24 ± 13.15	81.07 ± 13.99	0.273	0.785

## Data Availability

Data are available from the corresponding author on reasonable request.
